# Psychometric Evaluation and Misophonic Experience in a Portuguese-Speaking Sample

**DOI:** 10.3390/bs14020107

**Published:** 2024-01-31

**Authors:** Chloe Hayes, Jane Gregory, Rahima Aziz, Joaquim Cerejeira, Marina Cruz, José Augusto Simões, Silia Vitoratou

**Affiliations:** 1 Psychometrics and Measurement Lab, Biostatistics and Health Informatics Department, Institute of Psychiatry, Psychology and Neuroscience, King’s College London, London SE5 8AB, UK; chloe.1.hayes@kcl.ac.uk (C.H.);; 2 Department of Experimental Psychology, University of Oxford, Oxford OX2 6GG, UK; jane.gregory@linacre.ox.ac.uk; 3 Oxford Health Specialist Psychological Interventions Centre, Oxford Health NHS Foundation Trust, Oxford OX3 7JX, UK; 4 Faculty of Medicine, University of Coimbra, 3000-270 Coimbra, Portugal; jcerejeira@netcabo.pt; 5 Department of Psychiatry, Hospital do Divino Espírito Santo de Ponta Delgada, 9500-370 Ponta Delgada, Portugal; claudia.mc.cruz@azores.gov.pt; 6 Department of Medical Sciences, University of Beira Interior, 6201-001 Covilhã, Portugal; jars@uc.pt; 7 Center for Health Technology and Services Research (CINTESIS), 4200-450 Porto, Portugal

**Keywords:** misophonia, s-five, psychometrics, selective sound sensitivity syndrome, Portuguese, translation

## Abstract

Misophonia, a disorder characterised by an extreme sensitivity to certain sounds, is increasingly being studied in cross-cultural settings. The S-Five scale is a multidimensional psychometric tool initially developed to measure the severity of misophonia in English-speaking populations. The scale has been validated in several languages, and the present study aimed to validate the European Portuguese S-Five scale in a Portuguese-speaking sample. The scale was translated into Portuguese using a forward-backwards translation method. The psychometric properties of the S-Five scale were evaluated in a sample of 491 Portuguese-speaking adults. Confirmatory factor analysis supported a five-factor structure consistent with previous versions of the S-Five scale. The five factors were as follows: (1) internalising appraisals, (2) externalising appraisals, (3) perceived threat and avoidance behaviour, (4) outbursts, and (5) impact on functioning. The satisfactory psychometric properties of the S-Five scale further indicated its cross-cultural stability. As a psychometrically robust tool, the S-Five can measure misophonia in Portuguese-speaking populations, allowing future studies to explore and compare misophonia in this population.

## 1. Introduction

The literature has increasingly reported an inappropriate and disproportionate reaction to specific everyday sounds [1,2]. The complex disorder, termed misophonia, elicits intense physiological and emotional responses and is characterised by a highly decreased sound tolerance [3,4]. The trigger stimuli and the reactions experienced are widespread, varying by the individual; frequently reported organic sounds include eating, breathing, chewing, and nasal sounds. However, non-organic sounds (such as machine humming and clock ticking) have also been reported [1,5,6,7,8,9,10,11,12]. Tigger sounds tend to be pattern-based and repetitive [5,6,13,14], and reactions can be influenced by the context and the particular meaning that sound has to the individual [15,16]. Emotional reactions include anger, disgust [8,13,14,17], or anxiety and panic [12,18,19]. Some studies reported irritation as a primary response [8], while others found a negative association between misophonia severity and an irritation response to triggers [12,18,20]. Misophonia causes significant impairment in daily social and occupational functioning [15,19,21].

The development of measurement tools for assessing the severity of misophonia symptoms has been of large volume within the literature [1], but there remain limitations to such developed tools [1,2]. For instance, scale development has often occurred within non-representative samples and often fails to capture the complexities of misophonia in this advancing field [2]. Researchers in the area recognise the need for both screening and diagnostic tools that are psychometrically validated and adapted to all populations, including scale translation, to allow for cross-cultural understanding and comparisons.

The Selective Sound Sensitivity Syndrome Scale [12,20] is a 25-item scale for assessing misophonia symptom severity. A five-factor structure measures internalising appraisals (perception of oneself as a wrong or angry person for reacting to sounds), externalising appraisals (propensity to blame others for making the sound), emotional threat (sense of being trapped or helpless if unable to escape from sounds), outbursts (fear of or displays of aggressive outburst), and impact (current and future limitations in life from misophonia). The S-Five is supplemented by a trigger checklist (S-Five-T), which measures the nature of the emotional reaction (such as anger or disgust) to trigger sounds and the intensity of those reactions.

The initial psychometric evaluation was completed with a sample of individuals self-identifying as having the condition [20]. The five-factor structure, validity, and reliability have since been replicated in a UK general population sample [12] and also in samples using translated versions of the S-Five, including in Mandarin [22] and German [18].

To our knowledge, there is minimal data about misophonia in Portugal in the literature, with only case instances of misophonia currently being reported. One study in a Portuguese sample [23], in which a structured clinical interview was conducted, reported clinically significant misophonia in 25% of participants. However, the study was limited by a small sample size of 44 participants, and the clinical interview was based on numerous unvalidated misophonia scales. Therefore, to gain an accurate understanding of misophonia in a Portuguese-speaking population, there is a clear need for a psychometrically valid scale to be translated and evaluated in a large sample.

This study aimed to validate the S-Five in European Portuguese. For this purpose, we assessed the scale’s dimensionality, reliability (test-retest and internal consistency), and measurement validity within a Portuguese-speaking sample. In line with previous research, potential bias due to gender and age was also explored, as well as the association between misophonia and symptoms of depression and anxiety.

## 2. Materials and Methods

### 2.1. Recruitment

Participants were sampled through social media groups, mailing lists, adverts (social media groups sampled include Facebook (Misofonia Sindrome, transtorno obsessivo compulsivo), Reddit, posts on Twitter) (an article about misophonia in a Portuguese online newspaper called P3—Público was included), and contacts, and this type of convenience sampling allowed for sampling to be carried out quickly and at a low cost.

The consent of participants was collected before the completion of survey measures, with the provision of an information sheet (ethics approval reference HR-19/20-17173). The inclusion criteria set for participation in the study were being at least 18 years old and fluent in Portuguese. The exclusion of those diagnosed with a severe learning or intellectual disability, to the extent that participating in a self-report survey was impossible, was set as a criterion.

### 2.2. Measures

Demographic information from participants was collected, including age, gender, ethnicity, education level, occupation, country of birth, and country of residence. The survey asked respondents to report the formal diagnoses of mental health conditions (including disorders relating to mood, anxiety, trauma, psychosis, personality, eating, and substance abuse), audiological conditions (such as tinnitus), and neurodevelopmental conditions, whether they knew the term misophonia and if they identified with misophonia. Several self-report questionnaires were included, as described below.

#### 2.2.1. Selective Sound Sensitivity Syndrome Scale (S-Five) [20]

The S-Five consists of two parts. The first is the S-Five scale, a 25-item scale that measures the experience of misophonia (severity scale). The items are each rated on an 11-point interval scale from 0, “not at all true”, to 11, “completely true”. Previous validations [12,18,22] of the S-Five have identified five factors. The five factors capture aspects of externalising appraisals (for example, “people should not make certain sounds, even if they do not know about others’ sensitivities”), internalising appraisals (for example, “the way I react to certain noises makes me feel like I must be an unlikeable person deep down”), threat (“I feel trapped if I cannot get away from certain noises”), outburst (“some sounds are so unbearable that I will shout at people to make them stop”) and impact (“my job opportunities are limited because of my reaction to certain sounds”) of the disorder. The statements did not appear on the survey in any particular order.

The second part is the S-Five Trigger checklist (S-Five-T), which measures the frequency and intensity of a person’s reactions to trigger sounds. This study used the 37 trigger sounds from the original research [20]. For each trigger sound, a person responds to two questions relating to the past two weeks. First, a person selects the most prominent reaction they experience to the trigger, with the options of no feeling, irritation, distress, disgust, anger, panic, other feeling: negative, and other feeling: positive. Second, a person rates the intensity of their reaction from 0: does not bother me at all to 10: unbearable/causes suffering.

The triggers checklist can be scored to provide four indices: (1) trigger count (TC), which is the total number of triggers endorsed by a person; (2) reaction count (RC), the total number of times a person selected each of the possible reactions; (3) frequency/intensity of reactions score (FIRS), the sum of intensity scores for endorsed triggers; (4) relative intensity of reactions score (RIRS), an estimation of reaction intensity, relative to the number of endorsed trigger sounds.

#### 2.2.2. Translation Procedure

The S-Five was first translated independently from English to Portuguese by two authors, RA and MC, who are fluent in Portuguese and English. These two translations were compared to create an agreed-upon translation of the scale. This version was then back-translated to English by JC, fluent in Portuguese and English, and compared to the original S-Five to ensure the items were consistent. The A-MISO-S and MQ were also translated in this way. Please contact the corresponding author for these scales.

The original S-Five in English and the European Portuguese translation of the S-Five are presented in Appendix A.

#### 2.2.3. Other Measures

Several further measures were implemented in the sample to aid in the validation process of the European Portuguese translation of the S-Five.

Two misophonia scales were administered to assess the convergent validity of the S-Five: the A-MISO-S [17], a 6-item scale measure assessing the severity of one’s misophonia on a five-point ordinal scale (0–4), and the MQ [10], consisting of two subscales, rated on a five-point ordinal scale, with 19-items measuring sensitivity to sounds (MSYS) and emotional and behavioural responses to sounds (MEBS) and a single item interval scale, rating from 0 to 15, to assess the severity of misophonia (MSES). The A-MISO-S and MQ had excellent internal consistency, with alpha and omega values from 0.83 to 0.92.

The 9-item Patient Health Questionnaire-9 (PHQ-9) [24] is a commonly used measure of depression, previously translated and validated in a Portuguese-speaking population [25]. Items are rated on a 4-point ordinal scale with scores ranging from 0 to 27. Higher scores are indicative of greater depression symptoms. The 7-item Generalised Anxiety Disorder-7 (GAD-7) [26] was used to measure anxiety symptoms. Items are rated on a 4-point ordinal scale with a total score range of 0–21, with higher scores indicating more anxiety symptoms. The GAD-7 European Portuguese translated version used in this study was previously validated by Sousa and Viveiros [27]. The 18-item Anxiety Sensitivity Index-3 (ASI-3) [28,29] is a shorter version of the original Anxiety Sensitivity Index [30]. The scale, rated on a 5-point ordinal scale, measures fears about the possible consequences of anxiety sensations, including cognitive, physical, and social concerns.

### 2.3. Statistical Analysis

Confirmatory factor analysis (CFA) was implemented to assess the accuracy of the previously defined latent structure of the S-Five. The five-factor structure was compared to a unidimensional model. The multivariate normality (MVN) assumption of the S-Five items was evaluated using the MVN package [31] in R studio [32]. Five tests of multivariate normality were considered; for each, a non-significant (*p* > 0.05) test result provides evidence of the MVN assumption being met. The tests reported are Mardia’s multivariate kurtosis and skewness tests [33], Henze–Zirkler’s consistent test [34], Royston’s multivariate test [35], and Doornik–Hansen omnibus test [36]. The univariate normality of the S-Five items was also evaluated using Shapiro–Wilk’s test of normality. An appropriate estimator for CFA was used based on the results of the multivariate and univariate normality tests. To assess potential multicollinearity of the S-Five, items were correlated with each other to identify highly correlated items [37].

In factor analysis, a number of goodness-of-fit indices were considered in identifying the best-fitting latent variables model for the data. The guidelines set out by the ConPsy checklist [38] for adequate and close fit were followed. These indices and their criteria for close fit were the relative chi-square (χ^2^) with values less than 3 [39], the Root Mean Square Error of Approximation (RMSEA) with values < 0.05 [40], Standardised Root Mean Residual (SRMR) with values < 0.05 [41] and values > 0.95 for both the Tucker–Lewis Index (TLI) [42] and the Comparative Fit Index (CFI) [40]. For adequate fit to be concluded, we required a relative χ^2^ of less than five [40], an RMSEA < 0.10 [43], an SRMR < 0.08 [40,44], and values of CFI and TLI > 0.90 [40].

The values of two model selection indices, Akaike’s Information Criteria (AIC) [45] and Bayesian Information Criteria (BIC) [46], were reported for which lower values suggested a better model.

Measurement invariance, specifically scalar invariance, in the items of the S-Five, due to age and gender, was evaluated using the multiple indicators multiple causes model (MIMIC) [47,48]. Age and gender were included as exogenous variables in the MIMIC model to assess whether both the latent construct and its observed indicators exhibited consistent measurement properties across different ages and gender groups. Metric invariance, a foundational assumption in this analysis, ensures that the relationships between the latent construct and its indicators are equivalent across groups, allowing for meaningful comparisons of the underlying construct while controlling for potential variations in the scaling of observed variables.

The internal consistency of the S-Five, at scale level and within factors, was assessed using Cronbach [49] alpha (α) and McDonald [50] omega (ω), for which values > 0.7 are suggestive of satisfactory internal consistency [49,50]. Two additional estimates were considered for understanding the reliability of the factors: the alpha if the item was deleted, for which values lower than factor- or scale-level alpha are expected, and the item-total correlations (ITC: values between 0.3 and 0.8 required) [51]. Items falling outside of these bounds are suggestive of being problematic, with the content of such an item requiring further consideration.

To establish the convergent validity of the S-Five, the factor scales were correlated with A-MISO-S and the MQ subscales. A relationship between the S-Five and age, as well as differences in scores due to gender, were evaluated with non-parametric testing (Spearman’s rho and Mann–Whitney U tests).

The software Stata 17 [52] and Mplus 8 [53] were used for the analyses unless otherwise stated.

## 3. Results

### 3.1. Sample Characteristics

In total, 492 participants agreed to take part, of which one person was removed using listwise deletion due to missing items in the S-Five. Of the sample, N = 491, the mean age was 35 years old (mean = 34.6, SD = 11.0), ranging from 18 to 69 years old, and the majority of the sample identified as female (N = 394, 80%). From the final sample, 47% reported having misophonia, 23% did not identify as having misophonia, and 30% were unsure if they had misophonia.

Of 462 participants who responded to demographic and diagnostic questions, 93% were white or Caucasian (N = 429), and 79% were born in Portugal (N = 367). Regarding education, 44% (N = 205) held undergraduate degrees, and 40% (N = 183) held postgraduate degrees.

The most self-reported diagnoses were anxiety disorders, followed by mood disorders, with a reporting of 24% and 19%, respectively, of which the most frequently reported diagnoses were generalised anxiety disorder and depression. Other self-reported psychiatric diagnoses, grouped into general categories, were reported at a rate of between 6% and 3%, in the order of eating disorders, trauma-related disorders, personality disorders, and psychotic disorders. Audiological conditions were reported to affect 8% of the sample, and neurodevelopmental disorders were present for 6% of respondents.

### 3.2. S-Five Statements

#### 3.2.1. Statement Responses

For the majority of the S-Five statements, 19 of 25, “not at all true” was the most frequently selected response (endorsed by 21–56% of the sample). For the remaining six items, “completely true” was the most commonly selected response (with 17–33% of the sample endorsing the items). Four of these items are related to other people and sounds they may make, I06 “Others avoid making noises”, I13 “Others should not make sounds”, I21 “Others bad manners”, and I25 “Others disrespectful”; two items are related to feelings experienced when unable to avoid certain sounds, I07 “Feel anxious” and I10 “Experience distress”.

#### 3.2.2. Dimensionality and Measurement Invariance

The five tests of multivariate normality found the data to violate the normality assumption. The 25 items of the S-Five deviated from univariate normality according to the Shapiro–Wilk test (*p* < 0.001). The null hypothesis was rejected for all five multivariate normality tests (*p* < 0.001), suggesting a violation of the multivariate normality of the S-Five scores. Due to the violation of the normality assumption, factor analysis for continuous data was implemented using the maximum likelihood with a robust standard error estimator (MLR) [53]. The S-Five items did not suggest multicollinearity according to Spearman’s [54] rank correlation coefficients (r_s_ = 0.2–0.8).

The unidimensional model did not have an adequate fit to the data according to the goodness of fit indices (Rel χ^2^ = 7.9; RMSEA = 0.119 with a 90% CI (0.114, 0.123); CFI = 0.75; TLI = 0.73; SRMR = 0.078), and the factor loadings ranged from 0.46 to 0.87. The five-factor model had an improved fit over the one-factor model with an adequate fit in relation to the goodness of fit criteria (Rel χ^2^ = 3.2; RMSEA = 0.067 with a 90% CI (0.062, 0.072); CFI = 0.92; TLI = 0.91; SRMR = 0.05) and lower values for the model selection criteria. The loadings of the items to the factors were at least 0.67, indicating a suitable model fit (Table 1).

The MIMIC model was fitted to the data to explore the potential measurement bias of the S-Five. Five items (I14 “Avoid places”, I03 “Feel helpless”, I04 “Verbally aggressive”, I19 “Dislike self”, and I18 “Bad person inside”) were found to be measurement non-invariant with respect to age, adjusted for gender. However, for each of these items, the magnitude of the direct effect was very small, ranging from −0.03 to 0.03, interpretable as a change of 0.03 or smaller on the 0–10 scale of these items for a one-year increase in age.

Significant direct effects due to gender, adjusted for age, were found for four items of the S-Five. At the same levels of sound sensitivity, females scored higher on the following items by the relative effect: I23 “Shout at people” by 0.89 units, I07 “Feel anxious” by 0.51 units, I02 “Panic or explode” by 0.47 units, and I08 “Unlikeable person” by 0.43 units. However, the magnitude of less than 0.9 on an 11-point rating scale can be considered small (with medium to small effects). The remaining 21 items of the S-Five were measurement invariant with respect to gender. Thus, the S-Five can allow for the comparison of scores between different genders.

#### 3.2.3. Scores, Reliability, and Validity

The S-Five subscales and total scores were not significantly correlated with age (Table 2; *p* < 0.05). The threat score significantly differed between males and females, with females scoring, on average, 11 points higher (Table 2). The internal consistency was satisfactory for all S-Five factors (0.88 or higher; Table 2), with omega coefficients equal to that of Cronbach’s alpha. No items were identified as potentially problematic, with the item-total correlations ranging from 0.64 to 0.88 and a lower alpha if the item was deleted than the scale alpha for all items (Table 1).

The intercorrelations of the S-Five factors were moderate to strong (0.59–0.81; Table 3), and no significant correlations were found with age (Table 2). Evidence of convergent validity was found by correlating the S-Five with the subscales and total score of the MQ and the A-MISO-S total, for which moderate to high correlations emerged (0.46–0.86; Table 3). Moderate correlations were identified between all S-Five factors and the PHQ-9 and GAD-7 (rho ≤ 0.5; Table 3). Similarly, low correlations between the ASI-3 factors and total score and the S-Five were revealed (Table 3).

### 3.3. S-Five Trigger Checklist

#### 3.3.1. Reaction Counts

The descriptive statistics for the calculated reaction counts are shown in Table 4, which shows that no feeling had the highest average score, followed by irritation, then disgust.

**Figure 1:**
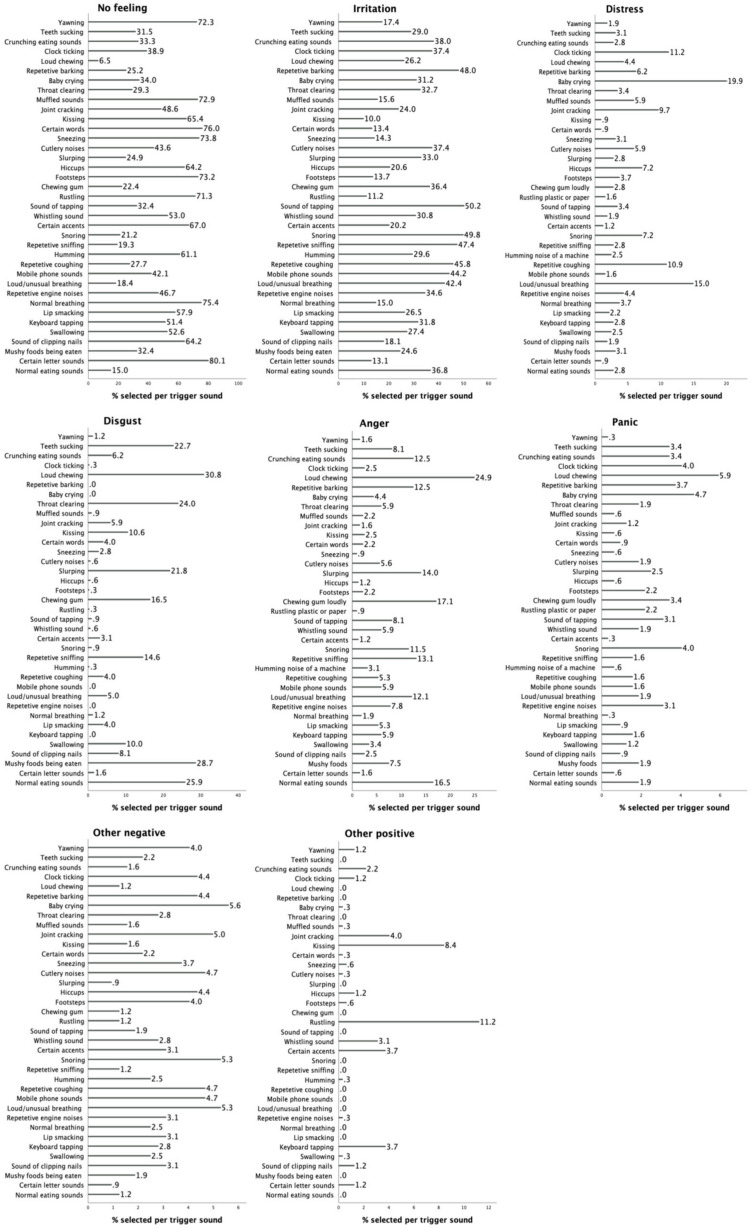
Percentage of participants selecting each type of reaction (no feeling, irritation, distress, disgust, anger, and panic) for the 37 trigger items.

Regarding each of the 37 trigger sounds, no reaction and irritation were the most frequently selected reactions for 36 sounds. For “loud chewing”, the most common reaction was disgust (31%), followed by irritation (26%) and anger (25%). For 20 of 37 sounds, the lowest reaction responses of no reaction and irritation captured 80% of the sample (Figure 1).

#### 3.3.2. Intensity

**Figure 2:**
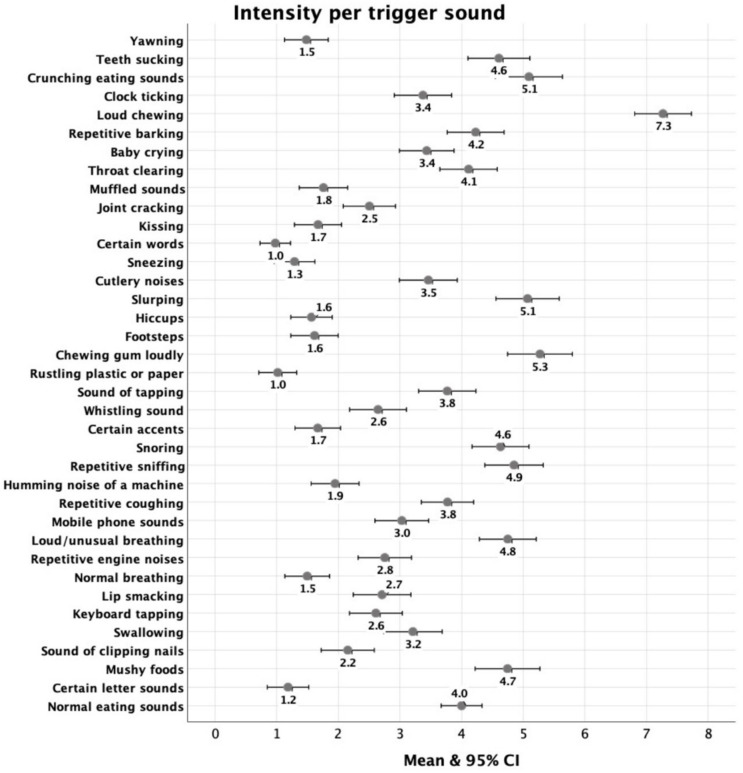
S-Five intensity score means and confidence intervals per trigger sound.

Regarding the intensity of the reactions reported, loud chewing, chewing gum, slurping, and crunchy eating sounds scored the highest (Figure 2). Certain letter sounds and words, rustling plastic or paper, and sneezing were the sounds that were reported with the lowest intensity of responses.

Table 5 shows the intercorrelations of the S-Five-T and the correlations of the S-Five-T with all the other measures. Low to moderate correlations were found between the S-Five-T scores and PHQ-9, GAD-7, and ASI-3. The reaction count for no feeling was negatively correlated with all other measures (*p* < 0.05) and had the strongest correlation with the trigger count, FIRS, and the MSYS of the MQ. The reaction count for anger had moderate to strong correlations with all of the misophonia scales used in the study (*p* ≤ 0.01), with the highest correlations seen between RC: anger and FIRS, S-Five total score, the MQ total score, and the MEBS of the MQ (rho > 0.6). The S-Five-T trigger count score and FIRS were found to have strong correlations with the MSYS of the MQ and the total MQ score.

## 4. Discussion

This study aimed to validate the existing 25-item S-five scale in the Portuguese-speaking population, showing that the S-Five, supported by its psychometric properties, is a reliable and useful measurement tool for misophonia. In collaboration with a Portuguese team experienced in the field of misophonia, the 25-item S-Five was accurately translated into European Portuguese whilst ensuring the psychometric integrity was maintained before distributing the survey to the Portuguese-speaking population. Factor analysis of the scale was carried out to assess the five-factor model, and the psychometrics properties were evaluated. This study produced novel findings that the S-Five scale is valid for measuring misophonia within the Portuguese-speaking population.

The five-factor solution of the S-Five scale, as shown in the original study [12,20] as well as in translation studies [18,22], was replicated in the Portuguese sample. The factors measure internalising appraisals, externalising appraisals, perceived threats, outbursts, and impacts on daily functioning. The S-Five was developed to measure these aspects of misophonia cross-culturally, and the present study has shown that the S-Five is reliable within a Portuguese-speaking sample, evidence that the scale can be used to assess the severity of misophonia within this population. This allows for the direct comparison of scores across the different cultures of which the S-Five has been evaluated [12,18,20,22]. In previous validations of the S-Five, the scale has been determined to be measurement invariant with regard to age and gender. While some items are non-invariant for gender, these effects have been of a small magnitude and, as such, considered negligible. Within the Portuguese-speaking sample, only one item from the outburst factor (I23 “Shout at people”) had a medium non-invariant effect; all other items had small negligible effects. This was further evidence that the S-Five is measurement invariant, allowing for scores to be compared across ages and genders.

The externalising factor had the highest average score of the five factors, and the impact factor had the lowest average score. This pattern was also seen in other cross-cultural studies that have used the S-Five to evaluate misophonia [12,18,20,22]. However, average threat scores were arguably higher within the Portuguese-speaking sample when compared to the UK general population [12] and the translation studies of the S-Five scale [22]. The UK misophonia population [20] was also found to have the highest average scores in the threat factor and the presence of a significant gender difference in threat scores. This could be explained by the higher percentage of participants self-identifying as having misophonia in this sample when compared to the previous validations of the S-Five [12,22], highlighting that those taking part in this study may not be representative of the general Portuguese-speaking population.

The S-Five factors had strong positive correlations with the MQ and A-MISO-S, in agreement with previous findings [12,20,22]. This further establishes that the S-Five has convergent validity in assessing misophonia severity within the Portuguese-speaking sample. Similarly, as found previously, moderate correlations between the S-Five, PHQ-9, and GAD-7 emerged. However, these correlations are likely due to the multidimensional nature of the S-Five, in comparison to alternative misophonia scales, as described in the literature [2,12], and the findings of an association between the symptoms of the three disorders [6,7,55,56,57]. While misophonia severity was moderately associated with anxiety symptoms, it was only weakly associated with anxiety sensitivity. Some studies have reported on the potential role of anxiety sensitivity in misophonia [58,59]. However, Wang and Vitoratou [60] proposed that this relationship could be explained by a shared overlap in anxiety symptoms rather than a unique contribution of anxiety sensitivity. More research is needed to investigate further the role of anxiety symptoms and sensitivity in misophonia.

This validation of the S-Five within the Portuguese-speaking population allows further investigations into misophonia, such as its prevalence, severity, and symptomology. The tool is also vital for both clinical and research utility, as the scale will allow for changes in misophonia to be reliably measured in response to treatments and interventions as these are developed and evaluated. The multidimensional structure and the flexible trigger checklist will allow for a more in-depth understanding of misophonia and the correlates of misophonia with other disorders and symptoms within this population.

There are several limitations of the present study that should not be overlooked. The main limitation is the exploratory nature of this study, which has used a mixed convenience sample that limits the ability to generalise to the target population. This restricts the application of the findings in establishing a cut-off score for clinically significant misophonia and cultural norms of misophonia. The sample consisted mainly of female participants, which may impact the interpretations that have been drawn from the study in relation to measurement invariance. Future research should seek a representative sample to confirm the results of the present study. Unlike previous studies, the current study does not specify the longevity of the S-Five in assessing misophonia severity. Thus, test–retest analysis would strengthen the scale’s reliability. Further, as the previous literature highlights, developing and implementing a structured clinical interview alongside the S-Five and other self-report measures would allow for discriminative validity of the S-Five to be established; this would ensure the measurement of misophonia in isolation from co-morbid conditions.

## 5. Conclusions

This study found that the five-factor structure of the S-Five was replicated in a Portuguese-speaking population, with proportionate evidence of reliability and validity. These findings highlight the robust nature of the existing S-five scale as a tool for measuring misophonia. While further evidence is needed to demonstrate the generalisability of the results to the general Portuguese population, this study provides preliminary findings that the S-Five can be administered in such a population for future research.

## Figures and Tables

**Table 1:** Confirmatory factor loadings for the five-factor structure of the S-Five measure (standardised) and item level reliability statistics.

Statements	Factor Loading	ITC	AID
Externalising			
I06 Others avoid making noises	0.67	0.67	0.87
I13 Others should not make sounds	0.72	0.73	0.86
I16 Others selfish	0.81	0.71	0.86
I21 Others’ bad manners	0.74	0.70	0.86
I25 Others disrespectful	0.91	0.79	0.84
Internalising			
I05 Respect myself less	0.68	0.64	0.91
I08 Unlikeable person	0.87	0.80	0.88
I12 Angry person inside	0.86	0.80	0.88
I18 Bad person inside	0.81	0.78	0.88
I19 Dislike self	0.83	0.79	0.88
Impact			
I01 Do not meet friends	0.72	0.71	0.91
I09 Eventually isolated	0.93	0.84	0.88
I14 Avoid places	0.82	0.80	0.89
I15 Cannot do everyday things	0.87	0.80	0.89
I20 Limited job opportunities	0.74	0.74	0.90
Outburst			
I04 Verbally aggressive	0.82	0.74	0.88
I17 Physically aggressive	0.80	0.78	0.87
I22 Violence	0.75	0.73	0.88
I23 Shout at people	0.80	0.75	0.88
I24 Afraid of outburst	0.81	0.74	0.88
Threat			
I02 Panic or explode	0.89	0.87	0.94
I03 Feel helpless	0.87	0.84	0.94
I07 Feel anxious	0.90	0.88	0.94
I10 Experience distress	0.89	0.86	0.94
I11 Feel trapped	0.91	0.89	0.94

Note: ITC: item-total correlation; AIC: alpha if item deleted.

**Table 2:** Descriptive statistics, Cronbach’s alpha coefficients, correlation with age and gender differences in scores of the total S-Five, and the factors of the S-Five.

Factor	Median (Q1, Q3)	Alpha	Correlation with Age (rho)	Gender Difference (Z)
Externalising	26 (14, 39)	0.88	0.06	−0.91
Internalising	14 (1, 29)	0.91	−0.07	0.70
Impact	6 (0, 19)	0.91	0.06	0.79
Outburst	11 (3, 24)	0.90	0.02	0.97
Threat	26 (6, 41)	0.95	−0.08	2.13 *
Total	91 (33, 140)	0.96	−0.01	0.80

Note: Q1, Q3: 25% quartile, 75% quartile; z: z statistic of the Mann–Whitney U test, * significant at *p* ≤ 0.05.

**Table 3:** Correlation coefficients (Spearman’s rho) of the S-Five statement subscales with other S-Five subscales, MQ, A-MISO-S, PHQ-9, GAD-7, and ASI3.

	Externalising	Internalising	Impact	Threat	Outburst	Total Score
S-Five (N = 491)						
Externalising	-					
Internalising	0.59 **	-				
Impact	0.59 **	0.75 **	-			
Threat	0.64 **	0.80 **	0.81 **	-		
Outburst	0.60 **	0.76 **	0.72 **	0.75 **	-	
Total score	0.79 **	0.89 **	0.87 **	0.92 **	0.87 **	-
Misophonia Questionnaire (N = 185)						
MSYS	0.46 **	0.61 **	0.61 **	0.62 **	0.66 **	0.68 **
MEBS	0.60 **	0.74 **	0.75 **	0.84 **	0.79 **	0.86 **
MSES	0.53 **	0.66 **	0.66 **	0.73 **	0.68 **	0.74 **
Total score	0.58 **	0.73 **	0.74 **	0.80 **	0.79 **	0.84 **
A-MISO-S (N = 214)						
Total score	0.56 **	0.73 **	0.72 **	0.79 **	0.65**	0.81 **
PHQ-9 (N = 440)						
Total Score	0.32 **	0.48 **	0.50 **	0.47 **	0.42 **	0.50 **
GAD-7 (N = 442)						
Total Score	0.35 **	0.50 **	0.49 **	0.49 **	0.46 **	0.52 **
ASI-3 (N = 216)						
Total score	0.23 **	0.31 **	0.31 **	0.35 **	0.30 **	0.35 **
Physical Concerns	0.17 *	0.24 **	0.29 **	0.36 **	0.25 **	0.30 **
Cognitive Concerns	0.25 **	0.31 **	0.35 **	0.34 **	0.34 **	0.37 **
Social Concerns	0.17 *	0.26 **	0.23 **	0.24 **	0.23 **	0.27 **

Note: * *p* ≤ 0.01; ** *p* ≤ 0.001; MSYS: Misophonia Symptoms Scale; MEBS: Misophonia Emotions and Behaviours Scale; MSES: Misophonia Severity Scale; A-MISO-S: Amsterdam Misophonia Scale; PHQ-9: Patient Health Questionnaire; GAD-7: Generalised Anxiety Disorder; ASI-3: Anxiety Sensitivity Index.

**Table 4:** Descriptive characteristics for the S-Five trigger checklist.

	N	Mean (SD)	Median (Q1–Q3)	Min–Max
RC				
No feeling	321	17.3 (7.4)	17 (12–23)	0–36
Irritation	321	10.8 (5.0)	10 (7–14)	0–27
Distress	321	1.7 (2.1)	1 (0–2)	0–12
Disgust	321	2.6 (2.6)	2 (0–4)	0–11
Anger	321	2.4 (3.4)	1 (0–4)	0–17
Panic	321	0.7 (1.7)	0 (0–1)	0–13
TC	200	20.8 (7.3)	22 (15–26)	4–37
FIRS	200	126 (70.0)	128 (72.5–172.5)	10–343
SIRS	200	5.7 (1.9)	6.2 (4.4–7.1)	1.2–9.3

Note: RC: reaction count; TC: trigger count; FIRS: frequency/intensity of reactions score; RIRS: relative intensity of reaction scores.

**Table 5:** Correlation coefficients (Spearman’s rho) of the S-Five triggers checklist with other S-Five subscales, MQ, A-MISO-S, PHQ-9, GAD-7, and ASI3.

	No Feeling	Irritation	Distress	Disgust	Anger	Panic	TC	FIRS	RIRS
S-Five-T									
No feeling	-								
Irritation	−0.53 **	-							
Distress	−0.32 **	−0.10	-						
Disgust	−0.30 **	−0.13 *	0.17 *	-					
Anger	−0.54 **	0.05	0.14 *	0.03	-				
Panic	−0.34 **	−0.12 *	0.19 **	0.10	0.35 **	-			
TC	−0.99 **	0.49 **	0.30 **	0.24 **	0.57 **	0.36 **	-		
FIRS	−0.85 **	0.35 **	0.23 **	0.18 *	0.66 **	0.40 **	0.88 **	-	
RIRS	−0.46 **	0.12	0.10	0.03	0.54 **	0.31 **	0.50 **	0.82 **	-
S-Five									
Externalising	−0.47 **	0.11	0.11	0.25 **	0.49 **	0.24 **	0.43 **	0.57 **	0.55 **
Internalising	−0.58 **	0.18 **	0.17 *	0.12 *	0.56 **	0.36 **	0.50 **	0.65 **	0.66 **
Impact	−0.58 **	0.17 *	0.23 **	0.12 *	0.52 **	0.43 **	0.56 **	0.69 **	0.65 **
Outburst	−0.54 **	0.23 **	0.16 *	0.12 *	0.57 **	0.32 **	0.47 **	0.64 **	0.66 **
Threat	−0.56 **	0.16 *	0.24 **	0.11 *	0.59 **	0.43 **	0.56 **	0.71 **	0.70 **
Total	−0.62 **	0.19 **	0.22 **	0.16 *	0.65 **	0.42 **	0.60 **	0.78 **	0.77 **
Age	−0.19 **	0.18 **	0.07	0.05	−0.01	−0.10	0.13	0.06	−0.03
Misophonia Questionnaire									
MSYS	−0.84 **	0.49 **	0.24 *	0.16	0.55 **	0.23 *	0.87 **	0.87 **	0.60 **
MEBS	−0.55 **	0.24 *	0.11	0.07	0.61 **	0.32 **	0.54 **	0.65 **	0.61 **
MSES	−0.47 **	0.25 *	0.22 *	0.12	0.50 **	0.18 *	0.43 **	0.50 **	0.50 **
Total score	−0.75 **	0.39 **	0.20 *	0.12	0.64 **	0.30 *	0.78 **	0.84 **	0.68 **
A-MISO-S Total	−0.61 **	0.22 *	0.14	0.18 *	0.56 **	0.39 **	0.59 **	0.72 **	0.65 **
PHQ-9 Total	−0.44 **	0.17 *	0.25 **	0.13 *	0.24 **	0.29 **	0.46 **	0.41 **	0.22 *
GAD-7 Total	−0.45 **	0.14 *	0.22 **	0.15 *	0.28 **	0.33 **	0.50 **	0.49 **	0.32 **
ASI-3									
Total score	−0.27 **	0.02	0.16 *	0.21 *	0.21 *	0.23 *	0.28 *	0.34 **	0.26 *
Physical Concerns	−0.16 *	0.01	0.10	0.12	0.17 *	0.17 *	0.18	0.29 *	0.28 *
Cognitive Concerns	−0.35 **	0.06	0.16 *	0.19 *	0.28 **	0.26 **	0.33 **	0.35 **	0.25 *
Social Concerns	−0.19 *	0.01	0.15	0.20 *	0.10	0.19 *	0.23 *	0.26 *	0.19

Note. * *p* ≤ 0.01; ** *p* ≤ 0.001; TC: trigger count; FIRS: frequency/intensity of reactions score; RIRS: relative intensity of reaction scores; MSYS: Misophonia Symptoms Scale; MEBS: Misophonia Emotions and Behaviours Scale; MSES: Misophonia Severity Scale; A-MISO-S: Amsterdam Misophonia Scale; PHQ-9: Patient Health Questionnaire; GAD-7: Generalised Anxiety Disorder; ASI-3: Anxiety Sensitivity Index.

## Data Availability

The data are freely available from Hayes, C. (2023). Psychometric Evaluation and Misophonic Experience in a Portuguese-Speaking Sample, 2023. [Data Collection]. Colchester, Essex: UK Data Service.

## Appendix A

The scoring guide for the S-Five scale and S-Five-T, translated to European Portuguese.

A. **The S-Five scale**

Por favor, leia cada declarcão cuidadosmente e baseie a sua reposta em como se sentem verdadeiros para consigo com base no seus pensamentos, experiências e reacções actuais. 0-Não é de todo verdade 10-Completamente verdade

**Exteriorização**

As pessoas não deveriam emitir determinados sons, mesmo que elas não saibam as sensibilidades dos outros

Eu sinto-me zangado com outras pessoas porque são desrespeitosas com os ruídos que fazem

As pessoas deveriam fazer todos os possíveis para evitar fazer ruídos que possam incomodar os outros

Eu reajo fortemente a certos sons porque não consigo aceitar como as pessoas possem ser tão egoístas, descuidadas ou mal educadas

Certos sons são apenas falta de educação, e não é estranho sentir uma raiva intense em relação a isso.

**Internalização**

A forma como reajo a certos ruídos faz-me sentir que no fundo eu devo ser uma pessoa dificil de gostar

O modo como eu reajo a certos sons faz-me pensar se no fundo eu não serei apenas uma má pessoa

Eu respeito-me menos a mim próprio(a) devido as minhas respostas a certos sons

Eu sinto que devo ser uma pessoa muito zangada por dentro pela forma como eu reajo a certos sons

Eu não gosto de mim próprio(a) nos momentos das minhas reações aos sons

**Impacto**

As minhas oportunidades de emprego estão limitadas devido **à** minha reação a certos ruídos

Eu não me encontro con amigos tão frequentemente como gostaria devido aos ruidos que eles fazem

Há locais onde eu gostaria de ir, mas não vou porque estou demasiado preocupado(a) sobre o impacto que os ruídos irão ter em mim

Prevejo que no futuro eu posso não conseguir fazer as tarefas do dia-a-dia devido às minhas reações aos ruídos

A forma como me sinto/reajo a certos sons ira acabar por me isolar e impedir-me de fazer tarefas do dia-a-dia

**Explosão**

Eu posso ficar tão irritado(a) com alguns ruídos que me torno fisicamente agressivo(a) com as pessoas de modo a fazê-las parar

Por vezes eu fico tão perturbado(a) com certos ruídos que uso a violência para os tentar parar

Alguns sons são tão insuportáveis que eu grito com as pessoas para fazê-las parar

Se as pessoas fizerem certos sons que não consigo suportar, torno-me verbalmente agressivo(a)

Eu tenho receio de fazer algo agressivo ou violento por não conseguir suportar o ruído que alguém está a fazer

**Ameaça**

Eu sinto-me encurralado(a) se não conseguir afastar-me de certos ruídos

Eu sinto-me ansioso(a) se não conseguir evitar ouvir certos sons

Se eu não me conseguir afastar de determinados ruídos, tenho receio que possa entrar em pânico ou sentir que vou exploder

Se não conseguir evitar certos sons, sinto-me desamparado

Eu posso sentir-me angustiado em conesquência de determinados ruídos

Todos os itens são classificados em uma escala ordinal de 0 a 10. Por favor, randomize os itens antes da administração.

**Pontuação**

Pontuações fatoriais e pontuação total: Adicione as respostas aos itens correspondentes para cada fator para calcular a pontuação fatorial e todos os itens para a pontuação total do S-Five. Cada fator possui 5 elementos, portanto as pontuações são diretamente comparáveis em termos de aprovação da declaração.

Faixa: As pontuações dos fatores variam de 0 a 50, a pontuação total varia de 0 a 250.

B. **The S-Five: Reactions (S-Five-R) ***
  (i) Pensando nas últimas semanas, qual é a principal sensação que este som lhe causou? (por favor escolha a mais característica) Nenhum Sentimento, Irritação, Angustia, Repugnância, Raiva, Pânico, Outro Sentimento Negativo, Outro Sentimento Positivo
  (ii) Pensando nas ultimas semanas, por favor avalie a intensidade da sua reacção a este som quando feito por outra pessoua ou objecto (de 0: não me incomoda nada até 10: insuportavel/sofrimento)

**Pontuação**

- **Emotional Reaction scores (ERS)** para cada emoção: contar em todos os gatilhos as vezes que cada emoção foi selecionada no(s) item(ns) para criar um índice para cada emoção (por exemplo, ERS-Anger, assumindo valores entre 0 e 37). O índice fornece informações sobre reações emocionais específicas entre os participantes.
- **Frequency/Intensity of Reactions Score (FIRS index)**: Adicione as respostas aos pontos (ii) em todos os gatilhos (intervalo de 0 a 370). O índice fornece informações combinadas sobre o número de sons de disparo e sua intensidade.
- **Trigger Count (TC):** Por favor, conte o número de respostas diferentes de zero para (ii). O índice assume valores entre 0 e 37 e fornece informações sobre o número de sons de disparo relatados por pessoa.
- **Trigger Relative Intensity (TRE):** Por favor, divida FIRS por TC para obter uma estimativa da intensidade das respostas de gatilho, em relação ao número de gatilhos relatados. O índice fornece informações sobre a intensidade da resposta aos gatilhos, independentemente de seu número.

* Lista de gatilhos atualmente incluídos no S-Five-R: Sons da mastigação, sons de certas letras, comida mole a ser mastigada, som de unhas a serem cortadas, engolir, bater nas teclas do teclado, estalar os lábios, respiração normal, sons repetitivos de motores, respiração anormalmente ruidosa, sons de telemóvel, tosse repetitive, cantar com os lábios fechados, fungar repetidamente, ressonar, certos sotaques, som de assobios, som repetido de bater levemente, crepitar, mascar pastilhas elásticas, passos, soluços, sorver, som de talheres, espirrar, certas palavras, beijar, estalar das articulações, sons abafados, limpar a garganta, choro de criança, ladrar repetitive, mastigação ruidosa, tiquetaque do relógio, sons ao mastigar comida crocante, sugar os dentes, bocejar
